# Classification of suicidal behavior calls in emergency medical services: a systematic review

**DOI:** 10.1186/s12245-023-00504-1

**Published:** 2023-04-17

**Authors:** Javier Ramos-Martín, M. Ángeles Contreras-Peñalver, Berta Moreno-Küstner

**Affiliations:** 1 Departamento de Personalidad, Evaluación y Tratamiento Psicológico. Universidad de Málaga, Doctor Ortiz Ramos, S/N 29010 Málaga, Spain; 2 Grupo Andaluz de Investigación Psicosocial (GAP) (CTS-945), Málaga, Spain; 3 Instituto de Biomedicina de Málaga (IBIMA), Málaga, Spain

**Keywords:** Suicide, Emergency medical services, Classification, Call centers, Systematic review

## Abstract

**Background:**

The aim of this systematic review was to examine the classification of calls for suicidal behavior in emergency medical services (EMS).

**Methods:**

A search strategy was carried out in four electronic databases on calls for suicidal behavior in EMS published between 2010 and 2020 in Spanish and English. The outcome variables analyzed were the moment of call classification, the professional assigning the classification, the type of classification, and the suicide codes.

**Results:**

Twenty-five studies were included in the systematic review. The EMS classified the calls at two moments during the service process. In 28% of the studies, classification was performed during the emergency telephone call and in 36% when the professional attended the patient at the scene. The calls were classified by physicians in 40% of the studies and by the telephone operator answering the call in 32% of the studies. In 52% of the studies, classifications were used to categorize the calls, while in 48%, this information was not provided. Eighteen studies (72%) described codes used to classify suicidal behavior calls: a) codes for suicidal behavior and self-injury, and b) codes related to intoxication, poisoning or drug abuse, psychiatric problems, or other methods of harm.

**Conclusion:**

Despite the existence of international disease classifications and standardized suicide identification systems and codes in EMS, there is no consensus on their use, making it difficult to correctly identify calls for suicidal behavior.

**Supplementary Information:**

The online version contains supplementary material available at 10.1186/s12245-023-00504-1.

## Background

According to the World Health Organization (WHO), suicide has emerged as one of the greatest public health problems worldwide, second only to traffic accidents as a cause of death in the population aged 15 to 29 years [1, 2].

Studies show that suicide attempts are the most important risk factor in the prediction of suicidal behavior [3]. Individuals with a previous history of self-harm are 25 times more likely to die by suicide than those who do not, and it is estimated that there are 20 previous attempts for every death by suicide [2–4].

Early diagnosis and treatment of these behaviors, as well as the adoption of appropriate measures to prevent the progression from suicidal ideation to death by suicide is one of the most effective preventive measures [4]. Accordingly, if the attention given to individuals who attempt suicide is swift, immediate, and appropriate, the number of suicides may be reduced. The risk of suicide can also be decreased with a suitable prevention and treatment program implemented by professionals in the prehospital and hospital setting [1, 5].

Studies show that contact between the population with suicidal behavior and health services is not sufficient to identify or prevent this health problem. Only 33% of people who die by suicide were hospitalized in the preceding year, which suggests that a high percentage of people received no medical or psychological help [6, 7]. Moreover, some people with suicide attempts only have contact with medical services through the emergency department [8–10].

EMS are among the first medical units to attend to people who have experienced severe events such as suicide attempts and are responsible for transferring patients to the emergency department [11–13]. The response of EMS to calls for suicidal behavior is crucial in order to improve medical care for this population [14]. Thus, proper identification and classification of calls can ensure that the individual is given access to psychiatric and psychological treatment to prevent future attempts with a fatal outcome [6, 15].

Since the detection of suicide attempts and related behaviors is necessary to prevent deaths, more studies are needed, especially in prehospital emergency services [16, 17]. With regard to suicidal behavior, its identification, diagnosis, and accurate recording are essential to provide a complete overview of the suicidal behavior. However, there is a lack of information on how suicidal behavior is recorded in the prehospital emergency setting. The aim of this systematic review was therefore to determine which classifications and codes are used in EMS to identify and record suicidal behavior, which professional assigns them, and when they are assigned during the process of attending an emergency call.

## Methods

### Study design

We performed a systematic review following the Preferred Reporting Items for Systematic Reviews and Meta-Analysis (PRISMA) statement [18]. The protocol was registered in the PROSPERO database (CRD42021227036).

### Search strategy

Two psychologist co-authors of this study (MAC and JR) independently conducted literature searches through PsycINFO, Pubmed, Scopus and Science Direct databases during the period September–October 2020. The search string was: (“emergency medical services” OR “emergency healthcare” OR “prehospital emergency” OR “911 calls”) AND “suicid*” in the title, abstract and keyword sections.

The following inclusion criteria were applied: a) empirical studies on suicidal behavior in emergency medical services (EMS); b) studies in Spanish or English; c) studies published between January 1, 2010 and October 31, 2020.

### Study selection

First, the two researchers reviewed the title and abstract of all the studies found through the literature searches to determine which met the inclusion criteria. After identifying potentially eligible studies, the full text of each study was reviewed. All studies that did not meet the inclusion criteria were excluded. The final decision concerning the included studies was made by discussion between the two authors and any disagreement was resolved by the third co-author of the study (BMK).

### Data extraction and variables

The variables collected from the different studies are detailed below and in Table 1.

- *Characteristics of the study variables*: country, geographic study area, population, data collection period, sources of extraction of the calls, total number of calls, number of suicidal behavior calls, and quality of studies.
- *Outcome variables*: moment of call classification, professional who assigned the suicide classification, and classification system and suicide codes used. Prehospital calls were recorded from information received by telephone at the emergency coordinating centers. First, the telephone operator or physician answering the call asked questions following a protocol established for each type of call, determined the priority level based on a triage system according to severity [19, 20], and assigned a classification code. Next, the professional who attended the patient on site classified the same call.

**Table 1:** Description of the variables collected from the studies

Variable	Description
*CHARACTERISTICS OF THE STUDIES*	
Country	Country in which the study was conducted
Geographic study area	Name of the geographic study area
Population	Number of inhabitants of the geographic study area
Data collection period	Time period in which data collection was performed
Sources of extraction of the calls	Databases from which the calls were extracted
Total number of calls	Sample of calls used in the study (N)
Number of suicidal behavior calls	Suicidal behavior calls extracted from total calls (N/%)
Quality of study	Indicates the quality range of the study through the Manual for Quality Scoring of Qualitative Studies expressed as a percentage
*OUTCOME VARIABLES*	
Moment of call classification	Point in time when codes are assigned to a call
Professional who classifies the calls	Person who classifies a call as suicidal behavior
Type of classification	Classification system used to categorize the calls
Suicide codes	Codes to classify calls as suicidal behavior, according to the classification used

Included studies were independently assessed for quality by two reviewers using the *Manual for Quality Scoring of Qualitative Studies* [21] (Table S1). Following Scott et al. (2014a), scores were transformed into percentages for a better understanding of quality. Accordingly, the minimum score corresponds to 0% and the maximum to 100%, with higher scores representing higher quality. Final quality scores are reported as the combination of the scores of each reviewer.

## Results

**Fig. 1:**
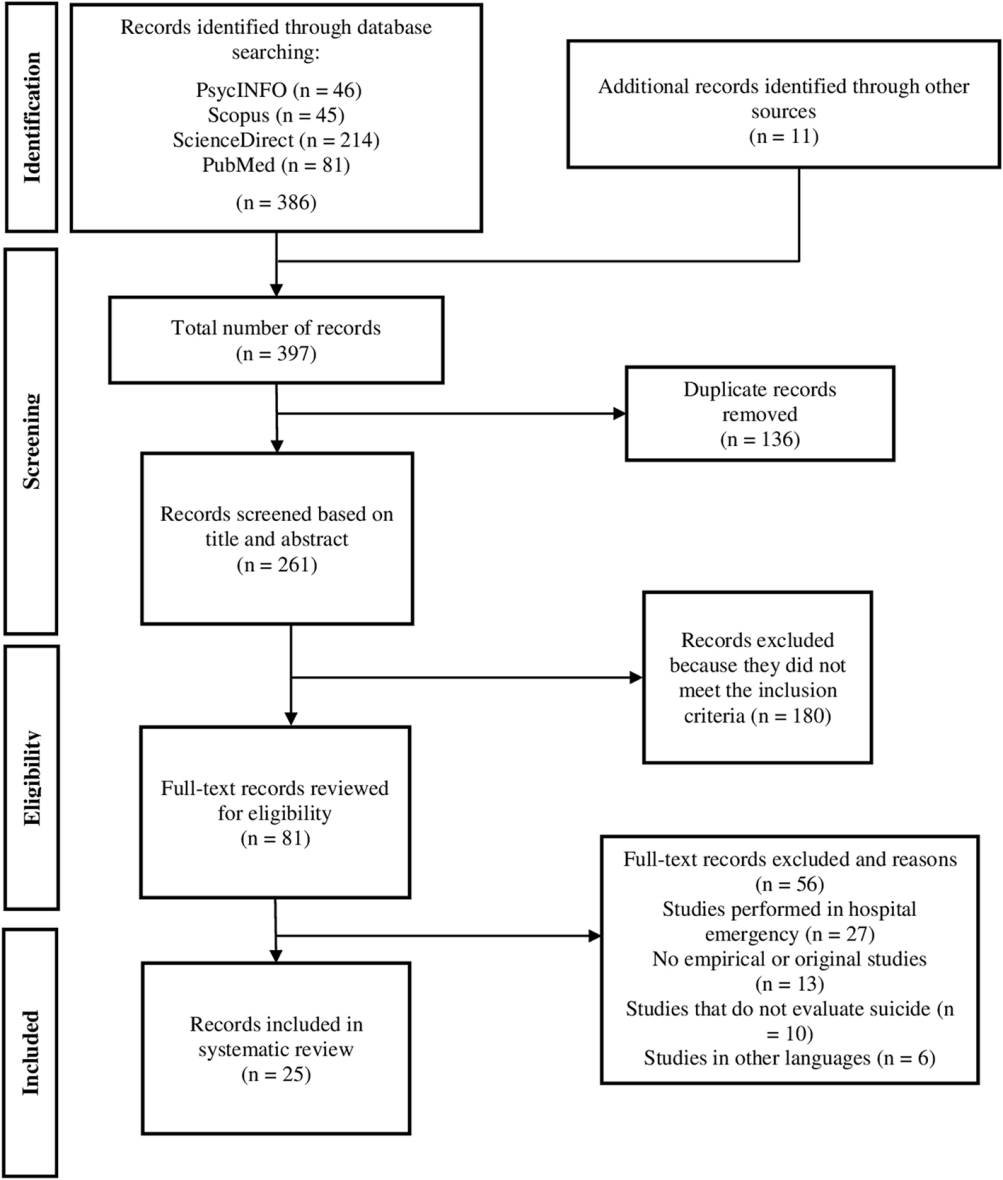
Flow chart of the study selection for systematic review

The search of electronic databases generated 386 results, and 11 studies were identified after manual bibliography searching. Thus, we found a total of 397 records. We excluded 136 duplicate articles, leaving 261 to be reviewed by title and abstract. Of these, 180 were excluded because they did not meet the inclusion criteria. The full text of the 81 remaining articles was reviewed, and 56 were excluded based on the exclusion criteria. Finally, a total of 25 publications were selected and included in the systematic review [10, 11, 19, 20, 22–42] (Fig. 1).

### Characteristics of the studies

The studies were conducted in various countries: Eight in Spain [10, 11, 20, 22, 25, 26, 31, 40]; three in the United Kingdom (UK) [28, 33, 41]; two in the United States of America (USA) [23, 29], Australia [19, 27], Brazil [24, 42], and Turkey [34, 35]; and one in Switzerland [30], Norway [32], Korea [36], Russia [37], France [38], and Japan [39]. According to the data collection period, two studies collected data for less than one year [33, 38] and fourteen for one year [11, 19, 20, 23–25, 28, 29, 31, 32, 34, 37, 41, 42]. Another study gathered data for two years [40], three for three years [35, 36, 39], one for six years [27], two for seven years [10, 22], and another two for ten years [26, 30]. According to the sources of extraction of the calls, thirteen studies used an EMS Coordination Center database [10, 11, 20, 22, 23, 25, 26, 31, 33–35, 39, 40], four used a hospital database [27, 28, 32, 38], and eight used ambulance records [19, 24, 29, 30, 36, 37, 41, 42]. Regarding the number of suicide calls, we observed great variability in the total number of calls selected by the studies, ranging from 779 to 6 million. With regard to the quality of the studies used in the systematic review, the quality was 90% in five studies, 80–85% in eight, 75–77.5% in nine, and less than 70% in the remaining three. Table 2 shows the characteristics of the included studies.

**Table 2:** Characteristics of the included studies

**ID-Article**	**First author (year-publication)**	**Country**	**Geographic study area (population)**	**Data collection period (month, year)**	**Sources of extraction of the calls**	**Total calls** **N**	**Suicidal behavior calls N (%)**	**Quality of study**^a^** (%)**
10	Mejías-Martín Y. (2018) [10]	Spain	Andalusia (8,464,411)	2007 – 2013	Coordination Center database	6,608,031	20,942 (0.31%)	90
11	Pacheco A. (2010) [11]	Spain	Spain (47,329,000)	2008	Coordination Center database	711,228	2.645 (0.4%)	52,5
19	Roggenkamp R. (2018) [19]	Australia	Victoria (6,695,000)	2015	Ambulance records	504,676	18,976 (3.7%)	82,5
20	Guzmán-Parra J. (2016) [20]	Spain	Malaga (1,528,851)	2008	Coordination Center database	163,331	1,171 (0.7%)	90
22	Mejías-Martín Y. (2019) [22]	Spain	Andalusia (8,464,411)	2007 – 2013	Coordination Center database	6,608,031	25,456 (0.4%)	90
23	Creed JO. (2018) [23]	United States	Wake County (1,112,000)	August 2013 – July 2014	Coordination Center database	1,555	101 (6.5%)	85
24	Ferreira TD. (2019) [24]	Brazil	Ribeirão Preto (674,405)	2014	Ambulance records	48,168	313 (0.6%)	77,5
25	Moreno-Küstner B. (2019) [25]	Spain	Malaga (1,528,851)	2014	Coordination Center database	181,824	1.728 (0.9%)	90
26	Celada FJ. (2018) [26]	Spain	Castilla La Mancha (2,035,505)	2006–2015	Coordination Center database	Not specified	1,308	77,5
27	Crossin R. (2018) [27]	Australia	Victoria (6,695,000)	January 2012 – June 2017	Hospital database	779	236 (30.3%)	77,5
28	Duncan EA. (2019) [28]	United Kingdom	Scotland (5,463,300)	2011	Hospital database	500,000	4,699 (0,9%)	85
29	Gratton M. (2010) [29]	United States	Kansas City (470,000)	2006	Ambulance records	72,668	734 (1%)	82,5
30	Holzer BM. (2012) [30]	Switzerland	Zurich (400,000)	May & June from 2001 through 2010	Ambulance records	4,239	135 (3.2%)	80
31	Jiménez-Hernández M. (2017) [31]	Spain	Malaga (1,528,851)	2008	Coordination Center database	163,331	1,380 (0.8%)	90
32	Johansen IH. (2010) [32]	Norway	Norway (5,367,580)	2006	Hospital database	5,672	8 (0.2%)	62,5
33	John A. (2016) [33]	United Kingdom	Wales (3,153,000)	December 2007 – February 2008	Coordination Center database	92,331	175 (0.2%)	85
34	Kayipmaz S. (2020a) [34]	Turkey	Turkey (83,154,997)	September 2018 – August 2019	Coordination Center database	Not specified	769	77,5
35	Kayipmaz S. (2020b) [35]	Turkey	Ankara (5,639,076)	January 2017 – June 2019	Coordination Center database	Not specified	6,777	77,5
36	Kim SJ. (2015) [36]	Korea	Korea (48,000,000)	2008 – 2010	Ambulance records	64,155	5,743 (8.9%)	67,5
37	Krayeva YV. (2013) [37]	Russia	Yekaterinburg (1,501,000)	March 2, 2009 – March 1, 2010	Ambulance records	2,536	984 (38.8%)	85
38	Maignan M. (2019) [38]	France	France (62,979,000)	March 17 / 18, 2015	Hospital database	Not specified	703	77,5
39	Matsuyama T. (2016) [39]	Japan	Osaka (2,669,000)	2010 – 2012	Coordination Center database	633,359	9,424 (1.5%)	82,5
40	Mejías-Martín Y. (2011) [40]	Spain	Granada (912,075)	2008 – 2009	Coordination Center database	149,570	535 (0.4%)	75
41	Scott J. (2014)[41]	United Kingdom	Yorkshire (5,234,700)	April 2010 – March 2011	Ambulance records	5,818	459 (7.9%)	77,5
42	Veloso C. (2018) [42]	Brazil	Teresina (893,246)	2014	Ambulance records	38,317	82 (0.2%)	77,5

^a^Range of available quality score was 0% to 100%. Final quality score reported as the combination of each reviewer’s scores

### Outcome variables

#### Moment of call classification

Seven studies (28%) classified the calls during the telephone call [20, 23, 29, 31, 38, 40, 41]; nine studies (36%) when the professional attended the patient at the scene [11, 24, 26–28, 30, 33, 37, 39], and five studies (20%) in both instances [10, 19, 22, 25, 32]. Four studies did not indicate when the call was classified (16%) [34–36, 42] (Table 3).

**Table 3:** Outcome variables

ID-Article	First author (year-publication)	Moment of call classification	Professional who classifies the calls	Type of classification	Suicide codes
10	Mejías-Martín Y. (2018) [10]	Answering the call Attending at the scene	Operator Physician	ICD-9 ICD-10	300.9 (Unspecified non-psychotic mental disorder (Suicidal Tendencies)) 305 [305.4, 305.8] (Nondependent abuse of drugs) 969 [96.9.0, .1, .2, .3, .4, .5, .8, .9] (Poisoning by psychotropic agents) E950-E959 (Suicide and self-inflicted injury) E980-E989 (Injury undetermined whether accidentally or purposely inflicted) V62.84 (Suicidal ideations) X84 (Intentional self-harm by unspecified means)
11	Pacheco A. (2010) [11]	Attending at the scene	Not specified	Not specified	Not specified
19	Roggenkamp R. (2018) [19]	Answering the call Attending at the scene	Nurses/Paramedics	MPDS	Not specified
20	Guzmán-Parra J. (2016) [20]	Answering the call	Operator	UECC Classification	Suicidal behavior: Self-injury and suicidal tendency, suicidal thoughts, suicide threat and suicide
22	Mejías-Martín Y. (2019) [22]	Answering the call Attending at the scene	Operator Physician	ICD-9 ICD-10	305 [305.4, 305.8] (Nondependent abuse of drugs) 969 (Poisoning by psychotropic agents) E950-E959 (Suicide and self-inflicted injury) E980-E989 (Injury undetermined whether accidentally or purposely inflicted) X84 (Intentional self-harm by unspecified means)
23	Creed JO. (2018) [23]	Answering the call	Operator	MPDS	Code 25 (psychiatric/suicide attempt)
24	Ferreira TD. (2019) [24]	Attending at the scene	Nurses	Not specified	Fatal or non-fatal suicidal behavior
25	Moreno-Küstner B. (2019) [25]	Answering the call Attending at the scene	Operator Physician	UECC Classification ICD-9	Suicidal behavior: Self-injury and suicidal tendency, suicidal thoughts, suicide threat and suicide E950-E959 (Suicide and self-inflicted injury) V62.84 (Suicidal ideations)
26	Celada FJ. (2018) [26]	Attending at the scene	Physician	ICD-9	E850-E858 (Accidental poisoning by drugs, medicinal substances, and biologicals) E860-E869 (Accidental poisoning by other solid and liquid substances, gases, and vapors) E950-E957 (Suicide and self-inflicted injury)
27	Crossin R. (2018) [27]	Attending at the scene	Paramedics	Not specified	Not specified
28	Duncan EA. (2019) [28]	Attending at the scene	Physician	AMPDS	09E03 (Hanging) 17D02J (Falls, Long fall (= > 6gy/2 m) – Jumper) 17D03J (Falls, unconscious or not alert – Jumper) 23 (Intentional poisoning) 25B01 (Psychiatric, serious hemorrhage) 25B02 (Psychiatric, minor hemorrhage) 25B03 (Psychiatric, suicide (threatening)) 25B04 (Psychiatric, jumper (threatening)) 25D01 (Psychiatric, not alert)
29	Gratton M. (2010) [29]	Answering the call	Operator	MPDS	Code 25 (psychiatric/suicide attempt)
30	Holzer BM. (2012) [30]	Attending at the scene	Paramedics	Not specified	Intoxication related to a suicide attempt
31	Jiménez-Hernández M. (2017) [31]	Answering the call	Operator	UECC Classification	Suicidal behavior: Self-injury and suicidal tendency, suicidal thoughts, suicide threat and suicide
32	Johansen IH. (2010) [32]	Answering the call Attending at the scene	Physician	ICPC-2	P77 (Suicidal behavior)
33	John A. (2016) [33]	Attending at the scene	Physician	Not specified	Suicidal ideation or intent
34	Kayipmaz S. (2020a) [34]	Not specified	Not specified	Not specified	Not specified
35	Kayipmaz S. (2020b) [35]	Not specified	Not specified	Not specified	Not specified
36	Kim SJ. (2015) [36]	Not specified	Not specified	Not specified	Not specified
37	Krayeva YV. (2013) [37]	Attending at the scene	Physician	Not specified	Circumstances of poisoning as: 2. Suicidal attempts
38	Maignan M. (2019) [38]	Answering the call	Physician	Not specified	Deliberation Self-Poisoning (DSP)
39	Matsuyama T. (2016) [39]	Attending at the scene	Physician	Not specified	Type of self-inflicted injuries: 1. Poisoning sleeping pill or tranquilizer 2. Poisoning by CO 3. Poisoning by other gas 4. Cutting and/or piercing wrist or arm 5. Cutting and/or piercing other part 6. Hanging 7. Jumping 8. Drowning
40	Mejías-Martín Y. (2011) [40]	Answering the call	Operator	ICD-10	X84 (Intentional self-harm by unspecified means)
41	Scott J. (2014) [41]	Answering the call	Not specified	AMPDS	Psychiatric/abnormal behavior/suicide attempt
42	Veloso C. (2018) [42]	Not specified	Not specified	Not specified	Not specified

*AMPDS* Advanced Medical Priority Dispatch System, *ICD-9* International Classification of Diseases, 9th revision, *ICD-10* International Classification of Diseases, 10th revision, *ICP-2* International Classification of Primary Care, 2nd edition, *MPDS* Medical Priority Dispatch System, *UECC* Urgencies and Emergencies Coordination Center

#### Professional who classifies the calls

Suicide codes were assigned by the following professionals: physicians in ten studies (40%) [10, 22, 25, 26, 28, 32, 33, 37–39], telephone operators in eight studies (32%) [10, 20, 22, 23, 25, 29, 31, 40], paramedics in three studies (12%) [19, 27, 30], and nurses in two studies (8%) [19, 24]. Four studies [10, 19, 22, 25] included calls in more than one professional category due to the classification being carried out by more than one professional. The six remaining studies [11, 34–36, 41, 42] did not specify who was responsible for assigning the codes (Table 3).

Regarding the moment at which the call was classified and the professional involved, as expected, all telephone operators classified the call during the telephone call [10, 20, 22, 23, 25, 29, 31, 40], while paramedics [27, 30] and nurses [24] did this when they attended the patient on site. In one of the studies [19], the calls were classified both during the call and when attending the incident at the scene, so more than one professional category was included. Concerning physicians, in two studies [32, 38] they classified the calls during the call, and in nine studies when attending the call on site [10, 22, 25, 26, 28, 32, 33, 37, 39] (Table 4).

**Table 4:** Moment of call classification, professional involved, and classification used

Moment of call classification	Article ID	Professional who classifies the calls	Classification
During the telephone call	10	Operator	ICD-9
	19	Paramedics/Nurses	MPDS
	20	Operator	UECC
	22	Operator	ICD-9
	23	Operator	MPDS
	25	Operator	UECC
	29	Operator	MPDS
	31	Operator	UECC
	32	Physician	ICPC-2
	38	Physician	Not specified
	40	Operator	ICD-10
	41	Not specified	AMPDS
Attending at the scene	10	Physician	ICD-10
	11	Not specified	Not specified
	19	Paramedics/Nurses	MPDS
	22	Physician	ICD-10
	24	Nurses	Not specified
	25	Physician	ICD-9
	26	Physician	ICD-9
	27	Paramedics	Not specified
	28	Physician	AMPDS
	30	Paramedics	Not specified
	32	Physician	ICPC-2
	33	Physician	Not specified
	37	Physician	Not specified
	39	Physician	Not specified

*AMPDS* Advanced Medical Priority Dispatch System, *ICD-9* International Classification of Diseases, 9th revision, *ICD-10* International Classification of Diseases, 10th revision, *ICP-2* International Classification of Primary Care, 2nd edition, *MPDS* Medical Priority Dispatch System, *UECC* Urgencies and Emergencies Coordination Center

#### Classifications used

In twelve studies the classification systems used to categorize suicidal behavior calls were not specified (48%) [11, 24, 27, 30, 33–39, 42], while in thirteen studies they were indicated (52%) [10, 19, 20, 22, 23, 25, 26, 28, 29, 31, 32, 40, 41]. Four of these thirteen studies (16%) used the International Classification of Diseases (ICD) [10, 22, 26, 40]. One study used the ninth revision (ICD-9) [26], another used the tenth revision (ICD-10) [40], and two used both [10, 22]: the ninth revision when answering the telephone call and the tenth revision when attending the patient at the scene. Five studies (20%) used a computerized system based on protocols, the Medical Priority Dispatch System (MPDS) [19, 23, 24] and its Advanced version (AMPDS) [28, 41]. The other two studies (8%) used a classification developed by the Urgencies and Emergencies Coordination Center of Andalusia (Spain) (UECC) [20, 31], and one study (4%) used the International Classification of Primary Care-2 (ICPC-2) [32]. One study (4%) used both the UECC classification, when responding to the telephone call, and the ICD-9 classification, when attending the patient on site [25].

When analyzing the classification and country of the study, we observed that the ICD and the UECC classifications were used in Spain, the MPDS in the USA and Australia, the AMPDS in the UK, and the ICPC-2 in Norway (Table 3).

In relation to the moment of call classification and the classification used, the ICD-9 was used in two studies during the emergency telephone call [10, 22] and in another two studies when attending the patient at the scene [25, 26]. The ICD-10 was used in one study during the telephone call [40] and in two studies when attending the emergency on site [10, 22]. The UECC Classification was used in three studies during the call [20, 25, 31], and the MPDS was used in three studies at this same point in time [19, 23, 29]. The MDPS was also used in one study when attending at the scene [19]. The AMPDS was used in one study during the emergency call [41] and in another when attending the patient at the scene [28]. Finally, in one study the ICPC-2 was used for assigning codes both during the call and at the scene [32] (Table 4).

#### Codes used to classify suicidal behavior calls

In eighteen studies (72%), codes used to classify the calls were indicated [10, 20, 22–26, 28–33, 37–41], while in the remaining seven studies (28%) this information was not provided [11, 19, 27, 34–36, 42].

To present the results, we grouped the codes into two broad categories: (1) those that explicitly specified suicidal behavior and (2) those that referred to poisoning, psychiatric problems, etc. and that are also often used to classify suicidal behaviors.

Specific suicidal behavior and self-injury codes were used in ten studies [10, 20, 22, 24, 25, 28, 31–33, 40], while codes for intoxication, poisoning, or drug abuse were used in eight studies [10, 22, 26, 28, 30, 37–39]. Codes for psychiatric problems were also used in five studies [10, 23, 28, 29, 41], and codes related to other methods of suicide or self-harm such as jumping or cutting were used in two studies [28, 39] (Table 3).

When considering only the specific codes for suicidal behavior and the classification that includes them, the most used codes were E950-E959 (suicide and self-inflicted injury), E980-E989 (injury undetermined whether accidentally or purposely inflicted) and V62.84 (suicidal ideation) of the ICD-9 [10, 22, 25, 26], and X84 (intentional self-harm by unspecified means) of the ICD-10 [10, 22]. Code 25 (psychiatric/suicide attempt) of the MPDS was used [23, 29] and code P77 (suicidal behavior) of the ICPC-2 [32]. In studies applying the UECC Classification system, the most used term was suicidal behavior (self-injury and suicidal tendency, suicidal thoughts, suicide threat, and suicide) [20, 25, 31].

Alternatively, some studies used codes for intoxication, poisoning or drug abuse, or psychiatric problems that do not specify whether they are suicidal. These include, for example, 305, 969, E980-E989 of the ICD-9 [10, 22] and E850-E859, E860-E869 [26]. When the AMPDS was used, a group of psychiatric codes were included [28]. In some cases, a suicide code was reported without indicating the classification [30, 37–39] (Table 3).

## Discussion

To our knowledge, this is the first systematic review to analyze studies on suicidal behavior in EMS in order to identify the classification systems and codes used as well as the professional assigning the codes and the moment at which they are assigned. The main finding of our study is that there is a wide variety of classification systems and codes used in the reviewed studies. Similarly, the description of the recording of information varied considerably both by the professional classifying the call and the moment at which it is collected. In addition, in many of the studies this information was not indicated.

### On the moment of call classification and the professional classifying calls

The results of this review show that in a higher proportion of the studies the assignment of classification and suicide codes is made by the physician at the time of attending the emergency on site, followed by a lower percentage of the studies in which the telephone operators perform this classification when answering the call. Considering that on many occasions certain emergencies, such as pill taking, poisoning, self-trauma, etc., may not be related to suicidal behavior, it is logical that the clinical judgment of the physician attending the person on site has greater weight in establishing whether the behavior is suicidal than the telephone operator, since the physician is better able to assess and analyze the situation and obtain more information about the intentionality of the act. However, the qualitative study by Blanco-Sánchez et al. (2018) indicates that there is disagreement as to who should establish whether the call is for suicidal behavior. While the telephone operators believe that the healthcare professionals at the scene should determine whether the call involves suicidal behavior, the healthcare professionals believe that their role is not to determine when an act was intentional, since their priority is to save the person's life, and they consider that the assessment of intentionality should be made later in the hospital services [43].

### On classifications and codes

We found that international classifications, such as the ICD-9 and ICD-10 [44, 45], and protocol-based computer systems, such as the MPDS and the AMPDS [46], are the most commonly used when recording calls for suicidal behavior. We also found an internally developed classification used in Andalusia (Spain), created by the UECC, which is based on 14 categories and does not follow any of the international classifications [20, 25, 31]. In addition, a classification used in primary care by family physicians, the ICPC-2, was used in the out-of-hospital setting [47]. The use of international classifications of diseases such as the ICD can help to standardize the collection of information and allow comparisons to be made. However, the use of the UECC classification, which is specific to a single autonomous community in Spain (Andalusia), prevents comparisons even within the country itself.

Another advantage of the international classifications cited above is that they have specific codes referring to suicide and self-injury that are used to record calls for suicidal behavior. Nonetheless, our results show that these calls are recorded using a wide variety of codes, many of which are not specific to suicide. Specifically, in addition to the codes specific to suicide, others are used that refer to intoxication, poisoning or drug abuse, mental health problems, and other methods of harm such as jumping or cutting. Although the existing literature indicates that the causes mentioned are closely related to suicidal behavior, it cannot be confirmed that all cases of intoxication, cutting, or jumping are suicidal behavior. Thus, in many studies, the calls for suicidal behavior could be overestimated.

Several studies also include in the group of calls for suicidal behavior those classified as intentional overdose/poisoning [10, 22, 26, 28, 30, 37–39]. This is because intoxication by drugs or noxious substances is a method frequently used in cases of suicide attempts [15, 48–50]. However, caution should be exercised when considering intoxication, poisoning, or drug abuse as suicidal behavior, as the lack of information on intentionality could be adding false positives to this group of calls. Several studies have shown that there is an increasing misclassification of suicides as poisonings [51–53], which would lead to misreporting of suicides.

The existing literature on suicide indicates that in order to consider a behavior or an associated act as suicidal (intoxication, jumping, cutting, etc.), there must be intent to die [54–56]. However, several studies indicate that in EMS the major barriers encountered by health professionals (physicians, nurses, or ambulance personnel) when classifying a call as suicide or attempted suicide are determining the intentionality of the act [40, 43], the absence of protocols, and insufficient training to manage this type of situation [57, 58].

We also note that the ICD-9 and ICD-10 are the most commonly used classifications. In cases referring to suicide, self-inflicted injuries, poisoning, and psychiatric problems, the codes used are standardized classifications and are usually the same. Nevertheless, in many instances, the codes used are not specific to suicidal behavior but also refer to psychiatric problems, suicidal behavior, or other methods of self-injury. This variability in the codes used may be explained by differences in the training received by the professionals and the information collected at the scene.

The intentionality of the act and the variety of methods for classifying suicidal behavior or self-injury highlight the complexity of the concept of suicide and can therefore lead to an underestimation or overestimation of suicidal behavior. There is a need for a thorough understanding of the characteristics of suicidal behavior and an awareness that there may also be risk behaviors that involve self-destruction of the person without deliberate intent to die, such as the abuse of drugs, alcohol, or other substances [51].

Finally, this systematic review shows that there is a lack of information concerning the classifications used to record calls for suicidal behavior, as they are not provided in half of the included studies. We cannot be certain whether this lack of information is due to the different studies not including these data or to the EMS not using specific classifications to record suicidal behavior, since it is very difficult to assess it accurately. Problems arising from the definition of suicidal behavior may affect the accuracy of data recording, which is consistent with the study by Miret et al. (2010), in which they observed that suicide attempts had very low recording rates in emergency department clinical reports [59].

Among the limitations of our systematic review, we highlight the exclusion of studies in languages other than Spanish and English, with the consequent loss of information on the subject. In addition, we did not include gray literature in our search. The variability and lack of information on the outcome variables are also limitations of our review. The included studies are framed in a transition period between the use of ICD-9 and ICD-10, which would explain the variability in the classifications for assigning calls for suicidal behavior. All this makes it very difficult to draw precise conclusions on the classification of calls for suicidal behavior in EMS.

In conclusion, the lack of consensus on the use of classifications and codes in EMS poses a problem in identifying and quantifying suicidal behavior. This may have a direct impact on the medical care received by this group of individuals. Therefore, improving the classification of suicidal behavior in the out-of-hospital setting by training professionals working in this area could improve care of suicide-related cases.

## Supplementary Information

**Additional file 1:** **TableS1.** Manual for the quality rating of qualitative studies.

## Data Availability

All data generated or analyzed during this study are included in this published article (and its supplementary information files).
