# Supplementary material for: Classification of suicidal behavior calls in emergency medical services: a systematic review
Source: Int J Emerg Med. 2023 Apr 17;16:27. doi: 10.1186/s12245-023-00504-1 (PMC10108483; doi:10.1186/s12245-023-00504-1)
Supplement: Supplementary file 1 — Additional file 1: TableS1. Manual for the quality rating of qualitative studies. [file 12245_2023_504_MOESM1_ESM.docx]

**Supplementary Material**

**Table S1.** Manual for the quality rating of qualitative studies

| **Question** | **Criteria** | **Description** |
| --- | --- | --- |
| **1.** Question/objective clearly described? | Yes | Research question or objective is clear by the end of the research process (if not at the outset) |
|  | Partial | Research question or objective is vaguely/incompletely reported |
|  | No | Question or objective is not reported, or is incomprehensible |
| **2.** Design evident and appropriate to answer study question? | Yes | Design is easily identified and is appropriate to address the study question |
|  | Partial | Design is not clearly identified, but gross inappropriateness is not evident; or design is easily identified but a different method would have been more appropriate |
|  | No | Design used is not appropriate to the study question (e.g. a causal hypothesis is tested using qualitative methods); or design cannot be identified |
| **3.** Context for the study is clear? | Yes | The context/setting is adequately described, permitting the reader to relate the findings to other settings |
|  | Partial | The context/setting is partially described |
|  | No | The context/setting is not described |
| **4.** Connection to a theoretical framework/wider body of knowledge? | Yes | The theoretical framework/wider body of knowledge informing the study and the methods used is sufficiently described and justified |
|  | Partial | The theoretical framework/wider body of knowledge is not well described or justified; link to the study methods is not clear |
|  | No | Theoretical framework/wider body of knowledge is not discussed |
| **5.** Sampling strategy described, relevant and justified? | Yes | The sampling strategy is clearly described and justified. The sample includes the full range of relevant, possible cases/settings (i.e., more than simple convenience sampling), permitting conceptual (rather than statistical) generalizations |
|  | Partial | The sampling strategy is not completely described, or is not fully justified. Or the sample does not include the full range of relevant, possible cases/settings (i.e., includes a convenience sample only) |
|  | No | Sampling strategy is not described |
| **6.** Data collection methods clearly described and systematic? | Yes | The data collection procedures are systematic, and clearly described, permitting and “audit trail” such the procedures could be replicated |
|  | Partial | Data collection procedures are not clearly described; difficult to determine if systematic or replicable |
|  | No | Data collection procedures are not described |
| **7.** Data analysis clearly described, complete and systematic? | Yes | Systematic analytic methods are clearly described, permitting and “audit trail” such that the procedures could be replicated. The iteration between the data and the explanations for the data (i.e., the theory) is clear – it is apparent how early, simple classifications evolved into more sophisticated coding structures which then evolved into clearly defined concepts/explanations for the data. Sufficient data is provided to allow the reader to judge whether the interpretation offered is adequately supported by the data |
|  | Partial | Analytic methods are not fully described. Or the iterative link between data and theory is not clear |
|  | No | The analytic methods are not described. Or it is not apparent that a link to theory informs the analysis |
| **8.** Use of verification procedure(s) to establish credibility of the study? | Yes | One or more verification procedures were used to help establish credibility/trustworthiness of the study (e.g., prolonged engagement in the field, triangulation, peer review or debriefing, negative case analysis, member checks, external audits/inter-rater reliability, “batch” analysis) |
|  | Partial | - |
|  | No | Verification procedure(s) not evident |
| **9.** Conclusions supported by the results? | Yes | Sufficient original evidence supports the conclusions. A link to theory informs any claims of generalizability |
|  | Partial | The conclusions are only partly supported by the data. Or claims of generalizability are not supported |
|  | No | The conclusions are not supported by the data. Or conclusions are absent |
| **10.** Reflexivity of the account? | Yes | The researcher explicitly assessed the likely impact of their own personal characteristics (such as age, sex and professional status) and the methods used on the data obtained |
|  | Partial | Possible sources of influence on the data obtained were mentioned, but the likely impact of the influence or influences was not discussed |
|  | No | There is no evidence of reflexivity in the study report |
